# Cancer-Induced Neuropathic Pain: Clinical Features and Treatment Options

**DOI:** 10.7759/cureus.99374

**Published:** 2025-12-16

**Authors:** Stefano Barbaro, Laura Lanotte, Ester Scapini, Antonio Zagaria, Pierdomenico Carone, Gennaro Gadaleta Caldarola, Maddalena Vurchio, Michele Debitonto

**Affiliations:** 1 Pain Therapy Unit, Ospedale Monsignor Dimiccoli, Barletta, ITA; 2 Oncology Unit, Ospedale Monsignor Dimiccoli, Barletta, ITA; 3 Pain Therapy Unit, Ospedale San Camillo De Lellis, Manfredonia, ITA

**Keywords:** cancer pain, cancer-related neuropathic pain, neuropathic cancer pain, opioid medication, pain adjuvant therapy

## Abstract

Cancer-induced neuropathic pain results from tumor or treatment-induced damage to the somatosensory system. Manifesting as burning, shooting, or electric-like sensations, it is challenging to control and can significantly reduce the quality of life. Effective management requires a multimodal approach, using adjuvant analgesics such as anticonvulsants or antidepressants alongside standard therapies. This condition often persists after cancer treatment and necessitates ongoing assessment to determine therapeutic response and adjust interventions for optimal patient comfort and function.

## Editorial

In clinical practice, neuropathic cancer pain (NCP) remains underestimated, and treatment is often delayed due to diagnostic challenges. It greatly diminishes the quality of life and overall well-being for those living with and after cancer. The mechanisms behind neuropathic pain in cancer patients are intricate and can include direct tumor involvement, compression or infiltration of nerves, nerve damage caused by chemotherapy or radiotherapy, or complications resulting from surgery. Various pain management approaches, such as medications, interventional analgesia, physical therapy, and complementary therapies, can help reduce neuropathic pain and enhance the patient’s comfort and quality of life.

Cancer patients frequently experience mixed-pain syndromes [1]. The location of the tumor, the disease's stage, exposure to anti-cancer treatments, and the intensity of cancer pain are not the same as how common it is in patients. Approximately 40%-60% of adult cancer patients experience moderate to severe cancer pain [2,3], and around 40% of cancer patients experience NCP [4]. NCP has a complicated etiology that includes direct nerve invasion or compression by solid tumors, neurological toxicity, chemotherapy, and radiation [5]. Strong opioids are effective in treating cancer pain [6], but there is a dearth of high-quality clinical trial data about the use of analgesics.

For the treatment of mild pain, weak opioids (such as codeine and tramadol) are used either alone or in conjunction with adjuvant analgesics. Strong opioids at modest doses may provide comparable pain relief with fewer adverse effects when used as an analgesic treatment for NCP. The use of powerful opioids for NCP is not well documented in high-quality randomized clinical trials (RCTs) [6]. There are no significant differences between morphine, oxycodone, and hydromorphone when taken orally, according to several European standards, and any of these medications can be used initially to treat moderate to severe cancer pain, including NCP. There is insufficient RCT data to support buprenorphine's effectiveness for NCP, consisting of small sample sizes or a lack of control groups. Methadone has a comparable analgesic effect to morphine, according to data from six low-quality trials included in a 2017 Cochrane Review of methadone for non-specific cancer pain [6]. The problem related to methadone that often limits its use despite its good analgesic properties is the risk of QT interval prolongation.

The complicated pathophysiology of NCP frequently necessitates the combined administration of neuropathic adjuvants and opioids [7]. The first-line adjuvant analgesics for individuals with NCP are gabapentin and pregabalin, together with the antidepressants duloxetine and amitriptyline [7]. When compared to non-neuropathic chronic musculoskeletal pain, some claim that gabapentin provides the best results for those with neuropathic pain [8]. In general, little high-quality data support the use of gabapentin or pregabalin to treat NCP [8]. The most frequently employed medications are tricyclic antidepressants (TCA) and serotonin-norepinephrine reuptake inhibitors (SNRIs). Duloxetine, an antidepressant drug of the SNRI class, is additionally proposed as an initial therapeutic approach for neuropathic pain, notably when it stems from cancer therapy [9], such as peripheral neuropathy induced by chemotherapy. Medical cannabis contains different mixtures of tetrahydrocannabinol (THC) and cannabidiol (CBD). However, the supporting data for its effectiveness in managing general cancer pain remain uncertain, and evidence is particularly limited in cases of NCP.

For individuals experiencing insufficient pain relief or encountering unbearable side effects, even with the best possible use of oral and injectable opioids and non-opioid drugs, the administration of local anesthetics, opioids, or clonidine directly into the spine should be taken into account [9]. Additional methods, such as spinal cord stimulation, may be suitable for selected patients with NCP, although the extent of pain reduction may be less significant compared with individuals suffering from non-cancer-related pain [10]. A comprehensive Cochrane review in 2012 identified three randomized controlled trials (RCTs) evaluating transcutaneous electrical nerve stimulation (TENS) for the relief of cancer pain in adults; however, the findings were inconclusive due to the lack of reliable data from the trials [11]. TENS may still be useful adjunctively, especially in palliative settings, despite limited RCT data.

Dealing with NCP effectively poses difficulties. Opioids are still essential for handling NCP, according to global guidelines. Additional care options include using established treatment strategies with suggested adjuvant antidepressants, anticonvulsants, and topical analgesics. More RCTs would be helpful to optimize this challenging clinical management.
